# Development of a System for Predicting Hospitalization Time for Patients With Traumatic Brain Injury Based on Machine Learning Algorithms: User-Centered Design Case Study

**DOI:** 10.2196/62866

**Published:** 2024-08-30

**Authors:** Huan Zhou, Cheng Fang, Yifeng Pan

**Affiliations:** 1 The School of Big Data and Artificial Intelligence, Anhui Xinhua University, Hefei, China; 2 Department of Neurosurgery, National Cancer Center/National Clinical Research Center for Cancer/Cancer Hospital, Chinese Academy of Medical Sciences and Peking Union Medical College, Beijing, China

**Keywords:** machine learning, traumatic brain injury, support vector regression machine, predictive model, hospitalization

## Abstract

**Background:**

Currently, the treatment and care of patients with traumatic brain injury (TBI) are intractable health problems worldwide and greatly increase the medical burden in society. However, machine learning–based algorithms and the use of a large amount of data accumulated in the clinic in the past can predict the hospitalization time of patients with brain injury in advance, so as to design a reasonable arrangement of resources and effectively reduce the medical burden of society. Especially in China, where medical resources are so tight, this method has important application value.

**Objective:**

We aimed to develop a system based on a machine learning model for predicting the length of hospitalization of patients with TBI, which is available to patients, nurses, and physicians.

**Methods:**

We collected information on 1128 patients who received treatment at the Neurosurgery Center of the Second Affiliated Hospital of Anhui Medical University from May 2017 to May 2022, and we trained and tested the machine learning model using 5 cross-validations to avoid overfitting; 28 types of independent variables were used as input variables in the machine learning model, and the length of hospitalization was used as the output variables. Once the models were trained, we obtained the error and goodness of fit (*R*^2^) of each machine learning model from the 5 rounds of cross-validation and compared them to select the best predictive model to be encapsulated in the developed system. In addition, we externally tested the models using clinical data related to patients treated at the First Affiliated Hospital of Anhui Medical University from June 2021 to February 2022.

**Results:**

Six machine learning models were built, including support vector regression machine, convolutional neural network, back propagation neural network, random forest, logistic regression, and multilayer perceptron. Among them, the support vector regression has the smallest error of 10.22% on the test set, the highest goodness of fit of 90.4%, and all performances are the best among the 6 models. In addition, we used external datasets to verify the experimental results of these 6 models in order to avoid experimental chance, and the support vector regression machine eventually performed the best in the external datasets. Therefore, we chose to encapsulate the support vector regression machine into our system for predicting the length of stay of patients with traumatic brain trauma. Finally, we made the developed system available to patients, nurses, and physicians, and the satisfaction questionnaire showed that patients, nurses, and physicians agreed that the system was effective in providing clinical decisions to help patients, nurses, and physicians.

**Conclusions:**

This study shows that the support vector regression machine model developed using machine learning methods can accurately predict the length of hospitalization of patients with TBI, and the developed prediction system has strong clinical use.

## Introduction

Traumatic brain injury (TBI) surgery increases the risk of certain complications, posttraumatic head injury complications that can lead to severe acute and chronic morbidity and mortality, and these complications are known risk factors for prolonged hospital stays [1-4]. In addition, the average length of stay after surgery for patients with traumatic brain trauma is an important indicator of the quality of medical management in neurosurgery departments and the overall level of care in this disease area and to some extent reflects the severity and complexity of the patient’s disease. In the current environment of insufficient supply of medical resources, it is important to predict the hospitalization time of patients with brain trauma in advance by technical means, and then combine the predicted hospitalization time with ward management methods to improve the bed turnover rate and medical service capacity, so as to improve the current situation of patients’ difficulty in hospitalization, reduce unnecessary medical expenses, and alleviate the medical burden of society [5-8]. Therefore, the purpose of this study is to develop a system for predicting the length of stay of patients with brain trauma based on clinical data from hospital medical records and machine learning algorithms to provide clinical decision support for physicians and reference for nurses to coordinate ward management in advance.

Given the complexity and diversity of clinical data after brain injury, complex statistical features such as multiple nonlinearities between different factors are prevalent [9-11]; the use of traditional multifactor logistic regression (LR) analysis methods alone cannot establish a predictive model for the hospitalization time of patients with early brain trauma, at which time different machine learning models are required for statistical analysis and prediction [12,13]. Convolutional neural networks (CNNs) and support vector machines are currently the mainstream prediction models for processing and analyzing and building complex statistical data that enable feature extraction and building prediction models for clinical data information. In this study, multiple machine learning models were developed to predict the hospitalization time of patients with brain trauma, and the advantages and disadvantages of prediction models based on different machine learning algorithms were explored through a comparative analysis research method, and the model with the best performance was selected. Finally, based on the optimal machine learning models, we develop a system that can be applied to clinical decision-making.

This paper describes the development of a system for predicting the length of stay of patients with craniocerebral trauma based on a machine learning algorithm, using a machine learning method that was retrospectively applied to analyze clinical data of patients with craniocerebral trauma from the Second Affiliated Hospital of Anhui Medical University, and predicting the length of stay of patients with craniocerebral trauma from this dataset. Our objectives were to:

1. prospectively predict the length of stay of patients based on clinical data and
2. develop a system that can be applied to clinical decision-making by means of an optimal prediction model.

## Methods

### Data Sources and Exclusion Criteria

Our dataset was obtained from a total of 1128 case records from the Neurosurgery Center of the Second Affiliated Hospital of Anhui Medical University. The decision to discharge a patient requires discussion in the treatment group and assessment by an experienced neurosurgeon superior before a decision can be made. Therefore, in order to ensure that the output of the developed predictive length-of-stay model was determined by the assessment of experienced neurosurgeons and was not influenced by the subjective desire of the patient’s family to abandon treatment or transfer to another department resulting in loss of follow-up, we set exclusion criteria for the medical records:

1. Automatic discharge of the patient at the request of the patient’s family to forgo treatment.
2. Serious injuries in other areas requiring transfer to the relevant department for further treatment.
3. Patients with previous experience of craniocerebral injury.

The exclusion criteria were developed and the data that met the exclusion criteria were removed from the model we developed through natural language processing (NLP) techniques. A total of 1001 patients were successfully enrolled, and by random splitting, we used 70% of the data (700 items) for training the model and the remaining 30% (301 items) for testing the performance of the model. In addition, we also collected clinical data from 111 patients at the First Affiliated Hospital of Anhui Medical University as external test data for external validation of the model.

### Ethical Considerations

This retrospective cohort study was approved by the Ethics Committee of the Second Affiliated Hospital of Anhui Medical University (S20210098). Participants or proxies signed the relevant informed consent forms within 24 hours of admission.

### Feature Matrix

Table 1 summarizes the input data features used to predict length of stay. We selected a total of 28 features recorded in the medical record system that were available in our dataset, and these data can be used to prospectively determine the length of stay of patients with craniocerebral trauma in a practical application. These data were selected because of the experience provided by previous studies.

**Table 1.** Input dataset features.

Name	Type	Unit	Data availability (%)
Age	Integer	Years	100
Gender	Male/female	—^a^	100
Hypertension	Boolean	—	100
Diabetes mellitus	Boolean	—	100
Alzheimer disease	Boolean	—	100
Coronary disease	Boolean	—	100
Chronic bronchus	Boolean	—	100
Arthrolithiasis	Boolean	—	100
Hypothyroidism	Boolean	—	100
Hyperthyroidism	Boolean	—	100
Personal history of tumor	Boolean	—	100
Cirrhosis	Boolean	—	100
Pancreatitis	Boolean	—	100
Hyperlipidemia	Boolean	—	100
Cerebral infarction	Boolean	—	100
Chronic obstructive pulmonary disease	Boolean	—	100
Hepatitis	Boolean	—	100
Poliomyelitis	Boolean	—	100
Tuberculosis	Boolean	—	100
			
Nephrotic syndrome	Boolean	—	100
Atrial fibrillation	Boolean	—	100
Mechanism of brain injury	Motor vehicle accident/all on the same plane/falling from height/injuries caused by heavy objects/none	—	100
Is there any loss of consciousness after injury?	Boolean	—	100
Glasgow Coma Index score for admission	Integer	—	100
Head CT^b^ examination on admission	Skull fracture/cerebral contusion/subdural hematoma/subarachnoid hemorrhage/intracranial pneumatosis	—	100
Brain surgery	Boolean	—	100
Intensive care treatment	Boolean	—	100
Complications during hospitalization	Bacterial infection/tracheotomy/anemia/gastrointestinal bleeding/liver function damage/electrolyte disorder/respiratory failure/abnormal coagulation function/thrombocytopenia/heart failure/peripheral facial paralysis/posttraumatic epilepsy/cerebrospinal fluid leak/acute coronary syndrome/none	—	100

a Not available.

b CT: computed tomography.

### Estimation of Missing Data

For the missing data in this study, the model method is used to complete them. We will predict the missing fields as target variables based on other existing fields to obtain the most probable complementary values. If the column with missing values is a numerical variable, the regression model is used to complete it. If it is a categorical variable, the categorical model is used to complete it. The steps of [14] modeling method are as follows.

1. Determine the variables (characteristic columns) that fill in the missing values.
2. Splitting the original dataset: split the original dataset into 2 subsets according to the variables that need to be filled with missing values: (1) without missing values: dataset_train; and (2) with missing values only dataset_pred)

1. Identify and test the correlation of the variables of interest: empirical analysis determines which attribute columns are correlated with the variables filled with missing values, and statistical analysis tools are applied to view the correlations between the selected attribute columns on the dataset_train dataset for validation.

1. Modeling and prediction: use the dataset_train dataset to build a linear regression model and apply the built model to estimate predictions for the missing variables in the dataset_pred dataset,

1. Merge and reduce datasets: merge and reduce the 2 subsets into 1 dataset to prepare the data for subsequent modeling.

### Model Establishment

#### Overview

In this study, we established 6 models: random forest (RF), CNN, support vector regression (SVR), multilayer perceptron (MLP), back propagation (BP) neural network, and LR, and compared the mean absolute percentage error (MAPE) and goodness of fit of the actual and predicted values to determine the optimal model for predicting the hospitalization time of patients with craniocerebral trauma in the system. The MAPE formula is shown in equation 1, where *n* is the sample size, $y_{i}^{`}$ is the predicted value, and $y_{i}$ is the true value.


(1) $$MAPE=\frac{100\%}{n}\sum_{i=1}^{n}|\frac{y_{i}^{\prime }-y_{i}}{y_{i}}|$$

#### Random Forest

The RF model was chosen because we considered the following advantages of the RF model [15]:

1. It can handle very high-dimensional data and does not have to do feature selection because the subset of features is chosen randomly.
2. After training, it is able to derive feature importance.
3. When creating an RF, an unbiased estimate of the generalization error is used, and the model generalizes well.
4. The trees are independent of each other during training, which makes training fast and easy-to-make parallelization methods.

#### Convolutional Neural Network

CNN is a class of feedforward neural networks that contains convolutional computation and has a deep structure, which is one of the representative algorithms of deep learning and has been widely used in various fields. Compared with traditional neural network algorithms, CNN has stronger modeling ability to extract effective feature data from the input relevant data and learn the internal structure of the feature data for better prediction [16-18]. We consider that CNNs have the following characteristics, which are suitable for application in this study:

1. CNNs have a weight-sharing network structure, which reduces the complexity of the network model and reduces the number of weights.
2. The data (including image data) can be directly used as the input of the network, avoiding the complicated process of feature extraction and data reconstruction in traditional algorithms.

#### Support Vector Regression

SVR is suitable for solving various regression prediction problems thanks to kernel functions and a few support vectors that play a decisive role and has achieved excellent prediction results [19]. Drucker et al [20] proposed a new regression technique based on the Vapnik support vector concept in 1996. SVR was compared with regression techniques based on regression trees and ridge regression performed in the feature space. Based on these experiments, it is concluded that SVR will be advantageous in high-dimensional spaces because SVR optimization does not depend on the dimensionality of the input space. Considering that the input data of this study have 28 dimensions, the SVR machine was chosen to predict the length of stay of patients with craniocerebral trauma.

#### MLP, BP Neural Network, and LR

In addition, 3 classical regression models, namely, MLP, BP, and LR, were developed for application in predicting the length of stay in patients with craniocerebral trauma. These 3 models are also the most common prediction models in the field of applied clinical informatics and have achieved good results in a large number of related studies [21-29].

### Training Set and Test Set Ratio

After establishing these 6 machine learning models, all valid original data samples are randomly disrupted and divided into a training set and a test set. In order to study the effect of different training set sample sizes on the modeling effect of different machine learning algorithms, the same rules are used to divide the training set and test set into 50%, 60%, 70%, 80%, and 90% of the total samples, and the MAPE of the test set samples is used to evaluate the model error.

### External Dataset Validation Model

Considering that the ultimate goal of this study is to apply the system to clinical decision-making and ward bed management, external validation is needed to demonstrate that the optimal model selected in this study has a strong generalization capability, that is, the ability to predict datasets other than the modeled data. The reason for external validation is that overfitting may occur during the modeling process, in which case the model predicts the modeled dataset well, but does not work well for other datasets (test set). Such a model is obviously of no application value. Therefore, the model designed in this study uses 111 data from the First Affiliated Hospital of Anhui Medical University for external validation, and once the accuracy of the external validation of the model meets the requirements of the system application, the model can be encapsulated into the system developed in this study.

### Predictive Modeling Pipeline

**Figure 1.**
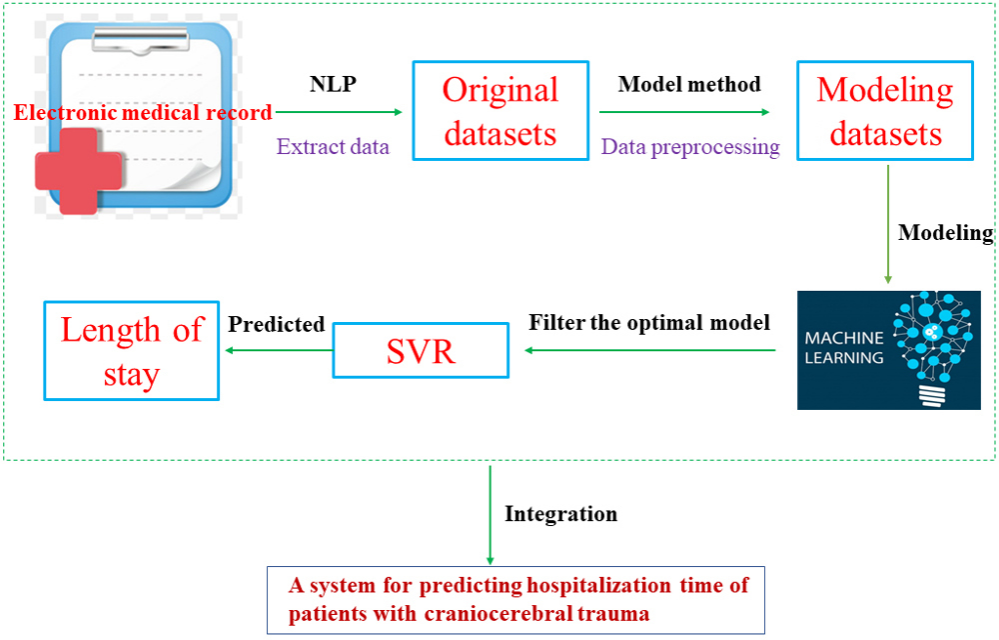
Development process of a system for predicting hospitalization time of patients with traumatic brain injury. NLP: natural language processing; SVR: support vector regression.

The development of our predictive modeling pipeline is based on Python 3.9 (Python Software Foundation), PyTorch 1.9.0 (Meta), and the django framework (Adrian Holovaty and Simon Willison), and consists of the following 4 main modules, and the process of developing the system is shown in Figure 1:

1. Data extraction module: Using NLP technology to extract the 28 features we need in the medical record system for patients with craniocerebral trauma, and based on the exclusion criteria we set, the data that meet the exclusion criteria are eliminated.
2. Data preprocessing module: The input feature data are automatically filled in with missing values using the model method to ensure the integrity of each patient record.
3. Model evaluation module: Visualize and evaluate the model-based predictions using the results of various standard key performance indicators.
4. Prediction of hospitalization time module: In this study, by comparing the prediction accuracy of the 6 models established, the model with the best prediction accuracy was established as the prediction model for system application, and finally the hospitalization time of patients with TBI was predicted by the input of the input features.

The predictive modeling pipeline ensures reproducibility and process stability and features many modules for processing data from medical record systems through machine learning algorithms to predicted length of stay data, which also supports the development of applied clinical systems for predicting the length of stay of patients with TBI.

## Results

### Proportion of Optimal Training and Testing Sets for Different Models

There is no fixed value for the partition ratio between the training set and the test set, and approximately 2 of 3 to 4 of 5 of the samples are usually used for training. The most common training set and test set ratios are 7:3 or 8:2. In order to achieve the best prediction accuracy of these 6 machine learning models designed in this study, the same rules are used to partition the training set and test set. The training set samples account for 50%, 60%, 70%, 80%, and 90% of the total samples. The MAPE of the test set samples was used to evaluate the prediction accuracy of these 6 models, and the experimental results are shown in Table 2.

The experimental results in Table 2 indicate that the optimal training and testing set ratios for different models may not necessarily be the same. Based on the experimental results, we selected the optimal training and testing set ratios for CNN, SVR, LR, RF, BP, and MLP as 0.7, 0.7, 0.8, 0.7, 0.8, and 0.7, respectively.

**Table 2.** Comparison of accuracy of 6 machine learning model test sets under different sample ratios of training sets.

Modeling set/test set	MAPE^a^ (%)					
	CNN^b^	SVR^c^	LR^d^	RF^e^	BP^f^	MLP^g^
0.5	30.76	28.46	40.57	32.74	35.93	33.70
0.6	24.83	22.78	35.14	25.63	28.65	26.49
0.7	12.19	10.69	27.68	13.47	21.37	18.41
0.8	15.63	18.25	22.75	15.40	19.28	21.76
0.9	28.43	26.71	34.13	26.24	32.17	28.43

a MAPE: mean absolute percentage error.

b CNN: convolutional neural network.

c SVR: support vector regression.

d LR: logistic regression.

e RF: random forest.

f BP: back propagation.

g MLP: multilayer perceptron.

### Model Accuracy

Divide all 1001 valid samples into training and testing sets. Divide the training and testing sets based on the results of the optimal ratio of the 6 models in Table 2. Then, start using these 6 algorithms to train the model for predicting hospitalization time, including cross-validation data. Repeat the training and testing 5 times and take the average value. For the prediction model of TBI patients’ length of stay, the goodness of fit and the MAPE of the test set are used to check the model performance. The reason for using the test set results to evaluate the model is that the test set results can screen out models with strong generalization ability for us, which is universal in our system. Based on this, we can directly adjust the parameters of different models through the errors of the model on the test set, making the predictive ability of the model better and the applicability of the system for predicting hospitalization time stronger.

From Table 3, we can see that in the test set, SVR has the lowest MAPE and the best goodness of fit, and CNN and RF perform well in the test set. The higher the error of LR, BP, and MLP than that of the other 3 models, the poorer the applicability of these 3 models, which should be considered for exclusion.

**Table 3.** MAPE and *R*^2^ of 6 models in the test set.

Model	MAPE^a^ (%)	*R* ^2^
CNN^b^	11.98	0.862
SVR^c^	10.22	0.904
LR^d^	21.83	0.718
RF^e^	14.27	0.827
BP^f^	18.35	0.785
MLP^g^	19.14	0.772

a MAPE: mean absolute percentage error.

b CNN: convolutional neural network.

c SVR: support vector regression.

d LR: logistic regression.

e RF: random forest.

f BP: back propagation.

g MLP: multilayer perceptron.

### External Dataset Validation Results

Considering that the data of the test set and training set in this study are from the medical record system of the Second Affiliated Hospital of Anhui Medical University, in order to avoid contingency of the model prediction accuracy, consider using the data from the medical record system of other hospitals to assist in verification. If the predictive performance of data input from other hospitals into the system is poor, consider developing different models for data from different centers to ensure the high accuracy of the system. If the prediction accuracy is excellent, it proves the universality of the system and can be applied more widely.

**Figure 2.**
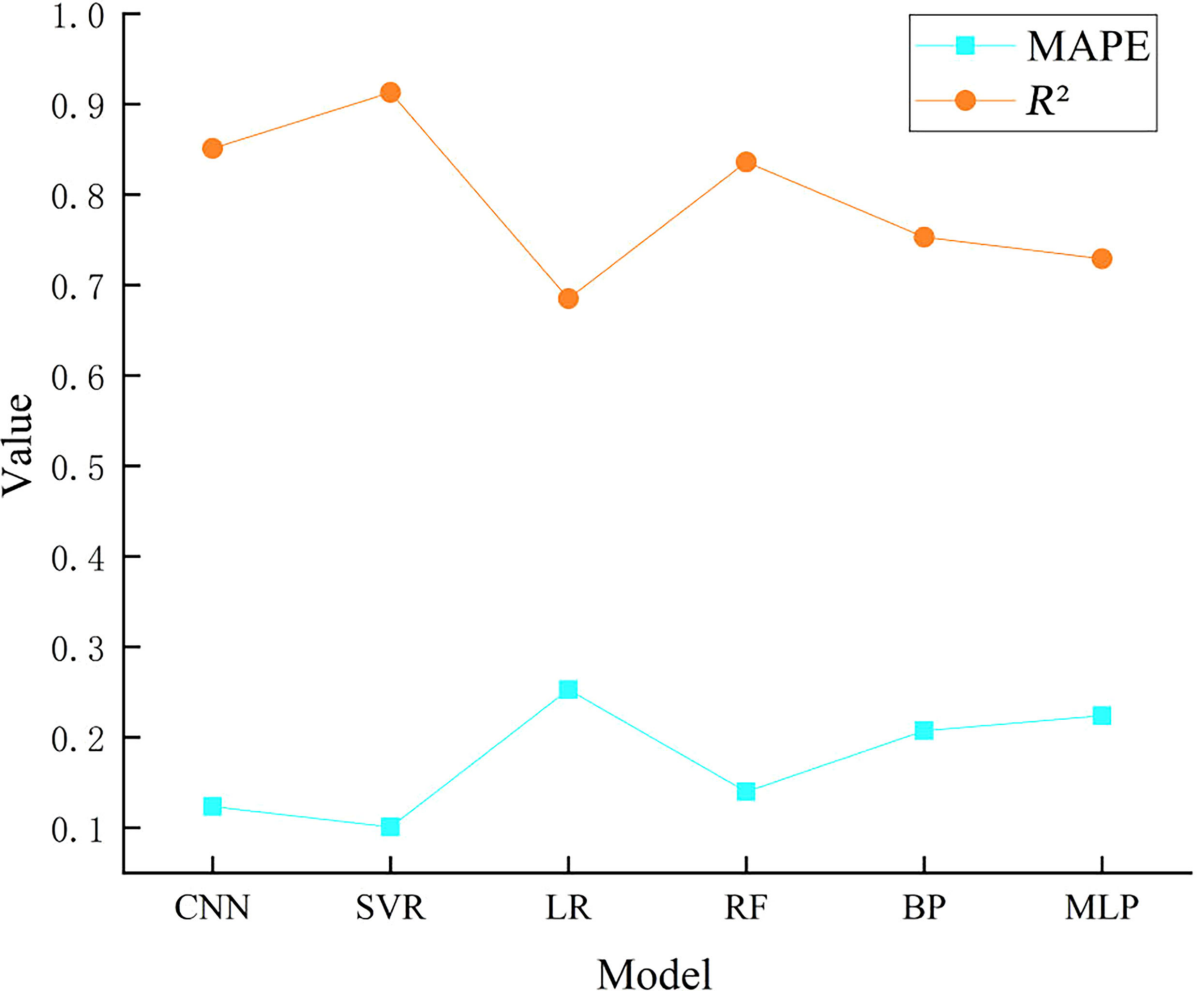
MAPE and *R*^2^ of 6 models in external test sets. BP: back propagation; CNN: convolutional neural network; LR: logistic regression; MAPE: mean absolute percentage error; MLP: multilayer perceptron; RF: random forest; SVR: support vector regression.

Therefore, in order to further compare the reliability of the algorithm, 111 data records from the First Affiliated Hospital of Anhui Medical University were used for external validation. A total of 28 types of input variables required by our model were extracted through NLP from the collected medical records and input into 6 models to compare the advantages and disadvantages of different models in the external dataset. The experimental results are shown in Figure 2. The experimental results show that the SVR-based hospital stay prediction model has the lowest error and the highest *R*^2^ in the external dataset, greatly maintaining the accuracy of the predicted data. It is the best machine learning model for predicting hospital stay. Therefore, the system we developed chose SVR as our prediction model.

### Satisfaction Evaluation of Patients, Nurses, and Doctors

After determining SVR as the final prediction model, this study ultimately designed a system to predict the hospitalization time of patients with TBI using data from an electronic medical record system. In order to verify the applicability of the clinical system, this study designed survey questionnaires for nurses and doctors to obtain satisfaction evaluations.

A total of 88 doctors and nurses from the First Affiliated Hospital of Anhui Medical University and the Second Affiliated Hospital of Anhui Medical University participated in the questionnaire. We set the following questions to assess health care workers’ satisfaction with the system:

1. Have you learned about and used our newly developed system for predicting length of stay for patients with TBI?
2. How well do you think the system supports clinical decision-making?
3. Do you think the system can improve your work efficiency?
4. How accurate do you think the system is?
5. Do you think the system’s predictions will help you better manage your patients’ length of stay?
6. Do you think the user interface of the system is user-friendly?
7. How well do you think the system protects data security and privacy?
8. Would you recommend the system to other colleagues or health care organizations?
9. Whether the system’s predicted outcomes will affect your treatment plan or patient management plan?
10. Do you feel the system integrates seamlessly with your existing workflow?

The above 10 questions will receive 1 point for agreement, 0 point for disagreement, and a maximum score of 10 points. The final average score is 9.18 points. The questionnaire results indicate that the system has strong practicality for patients. During the application process, most doctors and nurses stated that informing patients of the accurate length of stay during hospitalization can help alleviate their own stress, reduce fear of illness, and plan their life after discharge in advance.

## Discussion

### Principal Findings

The main finding of this study is that by using machine learning models, we can effectively predict the length of hospital stay for patients with TBI, which is of great significance for their rehabilitation and efficient use of medical resources. Specifically, the prediction system can help medical professionals more accurately evaluate the patient’s condition and develop treatment plans, thereby arranging surgery, medication treatment, and rehabilitation training reasonably; reducing waste of medical resources; and avoiding frequent hospitalization due to unstable patient recovery, reducing medical costs. In addition, predicting hospitalization time can also help patients and their families better plan their lives, understand treatment progress and rehabilitation plans, and improve confidence in treatment and rehabilitation outcomes. This study used a large amount of clinical data on TBI accumulated over the years at the Second Affiliated Hospital of Anhui Medical University, combined with machine learning algorithms, to develop a complete system from data extraction, preprocessing, and hospital stay prediction to model evaluation. This system realizes fully automatic operations from electronic medical records to hospital stay prediction and visualization. Users only need to query the hospital stay number to obtain the prediction results, which is convenient and fast to use and has high practicality. This research result not only provides assistance to doctors in clinical decision support but also significantly improves the rehabilitation effect and quality of life of patients.

In the 4 modules of system development, this study focuses on the prediction of hospitalization time module. We use NLP technology to extract enough clinical data from the electronic medical record for processing and screening, as the input variable of the model, and the length of stay as the output variable. Using input-output datasets, 6 machine learning models (CNN, SVR, LR, RF, BP, and MLP) were compared and constructed. In order to avoid the impact of the same proportion of training and testing sets on the prediction accuracy of different machine learning algorithms, and to achieve the best prediction accuracy of these 6 machine learning models designed in this study, the same rules were used to divide the training and testing sets into 50%, 60%, 70%, 80%, and 90% of the total samples and use the MAPE of the test set samples to evaluate the prediction accuracy of these 6 models. For these 6 models (CNN, SVR, LR, RF, BP, and MLP), we ultimately chose the optimal training and testing set ratios of 0.7, 0.7, 0.8, 0.7, 0.8, and 0.7, respectively. Divide the optimal ratio of training and testing sets and then start using these 6 algorithms to train models for predicting hospitalization time. Evaluate the performance of the prediction model based on the results of the testing set. The experimental results show that the minimum MAPE of SVR is 10.22%, and the best goodness of fit is 0.904. The CNN and RF perform well on the test set. The errors of LR, BP, and MLP are very high, and the goodness of fit is low, which indicates that these 3 models have poor clinical applicability, and should be excluded. At the same time, in order to further validate the reliability of the algorithm, the universality of the system for predicting hospitalization time of patients with TBI was verified through external datasets. The experimental results showed that the SVR-based hospitalization time prediction model had the lowest error and the highest *R*^2^ in the external dataset, maintaining the accuracy of the predicted data to a great extent. It is the best machine learning model for predicting hospitalization time. Therefore, the system we developed chose SVR as our prediction model.

Although in this study we compared and applied different machine learning algorithms to select the best algorithm SVR for application in the system and developed a system to predict the hospitalization time of patients with TBI through electronic medical records, the model adjustment and data-preprocessing steps were too specific, resulting in the current prediction mechanism being limited to local hospitals, while for hospitals in sparsely populated areas, it is not yet known whether the system has applicability. In addition, the differences in the electronic medical record systems of different hospitals can also lead to the inability to obtain the 28 input data required by this system through NLP, resulting in the inability to complete predictions or a decrease in prediction accuracy. Therefore, in the future, it is necessary to adopt a unified length of stay prediction framework to generate more reliable estimates and use them in different hospitals, where the system structure of electronic medical records for patient populations is similar. Finally, considering the inherent complexity and uncertainty of the system for predicting hospitalization time for TBI, as well as the relevant data currently being collected from different hospitals, we hope that the model needs to be widely applicable in subsequent system updates [30].

Nowadays, the application of machine learning models in the field of clinical decision support has become increasingly widespread [31-33]. However, this system can only predict the hospitalization time of patients with TBI. Therefore, more predictive indicators can be added in future research. In addition to hospitalization time, consideration should be given to increasing the treatment cost, readmission rate, rehabilitation time, and so on. Through these indicators, patients’ rehabilitation status can be more comprehensively reflected, helping doctors make better treatment decisions, and at the same time, precise medical plans can be formulated to provide better support and assistance for patients’ rehabilitation.

In addition to the fact that the system discussed above can only predict the length of hospital stay of patients with traumatic brain injury, the system itself still has certain limitations. First, the prediction accuracy of the system is too dependent on data quality, and the prediction accuracy of machine learning models depends on the quality of the data used. If the data quality is poor, it may affect the accuracy and stability of the model. Second, due to incomplete and insufficient data collection, there may be deviations in the data. This may affect the prediction results of the model and lead to misjudgment. Finally, machine learning models are often difficult to explain, which may lead to doctors not trusting the model’s prediction results. Therefore, in order to avoid data quality issues and biases, it is necessary to use multisource, multicenter, and diverse data as much as possible to train the model. In addition, data augmentation techniques can be used to expand the scale and diversity of the dataset, in order to improve the generalization ability of the model. In addition, to improve the interpretability of the model, interpretable machine learning techniques such as decision trees, rule learning, linear regression, and other models can be used to construct predictive models. At the same time, methods such as model visualization and feature importance analysis can be used to explain the predicted results of the model, enabling doctors to better understand the model’s decisions. In a word, to solve the limitations of the system, we need to combine domain knowledge and data analysis and mining technology to constantly optimize and adjust the model to meet the actual clinical needs.

### Conclusions

This study successfully developed a prediction model based on SVR by applying machine learning methods, which can accurately predict the hospitalization time of patients with TBI. This achievement not only demonstrates the strong potential of machine learning in the field of medical prediction but also provides strong support for clinical practice. By analyzing the medical data of patients in depth, the model can capture key factors that affect hospitalization duration and make accurate predictions based on them. This prediction system not only helps doctors better plan patient treatment and rehabilitation plans but also helps optimize the allocation of medical resources and improve the efficiency and quality of medical services. Therefore, the prediction system developed in this study has strong clinical practicality and is expected to become an important auxiliary tool for medical decision-making in the future, bringing patients a more personalized and efficient treatment experience.

## Abbreviations

BP: back propagation
CNN: convolutional neural network
LR: logistic regression
MAPE: mean absolute percentage error
MLP: multilayer perceptron
NLP: natural language processing
RF: random forest
SVR: support vector regression
TBI: traumatic brain injury
